# Method of Improved Fuzzy Contrast Combined Adaptive Threshold in NSCT for Medical Image Enhancement

**DOI:** 10.1155/2017/3969152

**Published:** 2017-06-28

**Authors:** Fei Zhou, ZhenHong Jia, Jie Yang, Nikola Kasabov

**Affiliations:** ^1^College of Information Science and Engineering, Xinjiang University, Urumqi 830046, China; ^2^Institute of Image Processing and Pattern Recognition, Shanghai Jiao Tong University, Shanghai 200400, China; ^3^Knowledge Engineering and Discovery Research Institute, Auckland University of Technology, Auckland 1020, New Zealand

## Abstract

Noises and artifacts are introduced to medical images due to acquisition techniques and systems. This interference leads to low contrast and distortion in images, which not only impacts the effectiveness of the medical image but also seriously affects the clinical diagnoses. This paper proposes an algorithm for medical image enhancement based on the nonsubsampled contourlet transform (NSCT), which combines adaptive threshold and an improved fuzzy set. First, the original image is decomposed into the NSCT domain with a low-frequency subband and several high-frequency subbands. Then, a linear transformation is adopted for the coefficients of the low-frequency component. An adaptive threshold method is used for the removal of high-frequency image noise. Finally, the improved fuzzy set is used to enhance the global contrast and the Laplace operator is used to enhance the details of the medical images. Experiments and simulation results show that the proposed method is superior to existing methods of image noise removal, improves the contrast of the image significantly, and obtains a better visual effect.

## 1. Introduction

Computed tomography (CT) images and Magnetic Resonance Imaging (MRI) images are extensively used in the detection of Cerebrovascular Accidents [1]. The quality of MRI imagery has made it an indispensable part of modern medicine; the quality of MRI imagery also directly influences the accuracy of doctors' diagnoses and treatments. Medical images often experience interference from factors such as noises and artifacts that may degrade the quality of the obtained image. Additionally, noise and artifacts introduce uncertainties to the medical image in the form of vagueness in the homogeneity of image segments or vague contrast between the object and background. This makes it difficult to segment or detect the contours and textures of an image. Image enhancement is one of the main concerns of image processing. At the present time, there are many methods of image enhancement, which can be classified into two principal groups: spatial domain-based methods and transformation domain-based methods. The widely used histogram equalization- (HE-) based methods are a form of spatial domain-based methods [2]. The resulting image has a uniform distribution of intensity after equalization, but these methods tend to overenhance the contrast if there are high peaks in the histogram, which can result in a harsh and noisy appearance of the resulting image. Numerous approaches for modifying histogram based contrast enhancement have been proposed in order to limit the level of enhancement. These modifications include automatic image equalization using Gaussian mixture modeling [3], power-constrained contrast enhancement based on histogram equalization [4], and entropy maximization histogram modification for image enhancement [5]. The retinex theory was originally proposed by Land and is based on the human visual system [6]. The retinex can provide color constancy and dynamic range compression [7]; however, the multiscale retinex algorithm creates a halo phenomenon in high contrast areas. With the development of multiresolution analysis, many methods for image enhancement in the transform domain are applied including the wavelet, curvelet, and contourlet methods. Curvelet transformations are able to best represent salient image features like edges and details [8]. The construction of the curvelet is not directly built into the discrete domain, so curvelet transformations fail to achieve an optimal nonlinear approximation for a higher order canonical singular edge. Scratches appear at the edges of the image after image processing. A 2-dimensional discrete wavelet transformation is extended from the one-dimensional function; therefore, it can only capture limited directions, and it is not able to effectively represent the directional information of the image. Contourlet transform is proposed in order to overcome this shortcoming of wavelet transform [9]. However, contourlet transform is limited in shift-invariance because of the downsampling process in the Laplacian pyramid and directional filter bank, and this drawback may cause pseudo-Gibbs phenomena around singularities, which reduces the local information and weakens the features of the directional selection. da Cunha et al. [10] proposed the nonsubsampled contourlet transform (NSCT), which has the characteristic of shift-invariance so that each pixel of the transform subbands corresponds to that of the initial image in same spatial location. It can isolate the noise from texture effectively, while refraining from the interference of new noise. Sajjadi et al. [11] put forward a set of simpler and more efficient filters for nonsubsampled pyramid in NSCT and introduce an improved method for modification of directional subbands' coefficient, which remove the noise while retaining a large amount of detail information. However, the textures and edges of the processed image will be blurred. Liu et al. [12] proposed a method in NSCT domain combining unsharp masking, which can highlight the local region of texture information, but the overall contrast has not been improved. Pal and King first employed fuzzy sets for image enhancement in 1981 and they received satisfactory results [13]. Wang and Yan [14] have designed an image enhancement algorithm based on improved fuzzy domain, in which an improved fuzzy shrink function was used to conduct denoising, but the process of this algorithm is complex so that it will bring difficulty to its application. Hasikin and Isa [15] proposed a fuzzy technique to enhance low contrast images where S membership function was used and the parameters in the membership function were computed using entropy and the index of fuzziness. Liu et al. [16] presented a method based on intuitionistic fuzzy sets theory, in which the restricted equivalence function is used to find the membership function to avoid the defect of Pal-King's algorithm and the new fuzzification, hyperbolisation, and defuzzification function are used to deal with the object and background areas of the image in parallel, but the problem of averting the amplification of noise and highlighting the textures and details at the same time has not been solved. Wang et al. [17] designed a medical image enhancement method based on improved fuzzy contrast and adaptive threshold, but the mean membership function is included in the fuzzy contrast, which makes the algorithm more complex, and the fuzzy contrast value would be not accurate. A constant is used to replace the mean function, which makes the calculation simpler, and the traditional threshold function has better denoising effect in this paper.

The Laplacian filter is an isotropic filter which has been commonly used as an enhancement operator to enhance image details. Adaptive threshold function is adopted to distinguish image details and noises in high-frequency subbands of the NSCT domain, which is used to remove noises. This paper proposes an algorithm, based on the above analysis, that combines the adaptive threshold function based on NSCT and the improved fuzzy contrast enhancement. The adaptive threshold is implemented on high-frequency subbands to eliminate the noise in order to avoid the amplification of the noise when enhancing the edges and details. The core contribution lies in the improved fuzzy contrast enhancement, which can dispel the quantization error in small neighborhood and reduce the loss of the details in large neighborhood. The combination of these methods can reserve more details of the initial image and thus increase the quality of the enhanced image. Experiments demonstrate that images produced by the proposed enhancement method are observed to be superior to the images obtained from other comparative enhancement algorithms.

## 2. Material and Methods

### 2.1. Figures, Consent, and Permissions

The figures used in this study were taken from several medical image resources mentioned in the acknowledgement section. The experimental protocol of this study was reviewed and approved by the College of Information Science and Engineering, Xinjiang University. After fully carrying out the procedure of the method proposed in this study, all study subjects signed an informed consent form. All methods in this study were strictly executed in accordance with the approval guidelines and regulations.

### 2.2. Theoretical Analysis

#### 2.2.1. The Theory of NSCT

**Figure 1:**
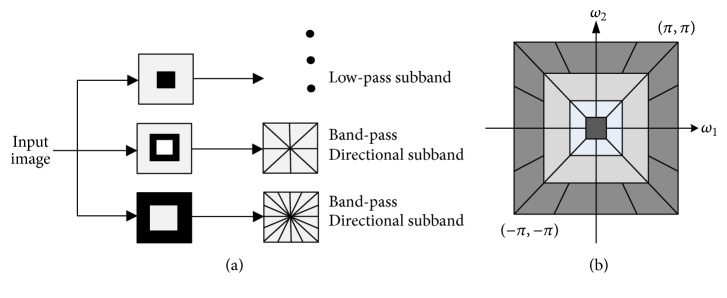
(a) NSCT structure and its decomposition process. (b) Idealized schematic diagram of frequency resolution.

NSCT is an overcomplete transformation which has shift-invariant, multiscale, and multidirection features [18]. The NSCT is built upon a 2D nonsubsampled pyramid and nonsubsampled directional filter bank (NSDFB). The nonsubsampled pyramid can be obtained by removing the downsamplers and upsamplers in the Laplacian pyramid, which decides the multiscale decomposition of the NSCT. The NSDFB is constructed by eliminating the downsamplers and upsamplers in the directional filter bank, which decides the directional decomposition of the NSCT. The NSCT can suppress the pseudo-Gibbs phenomena effectively, capture the intrinsic geometrical structures accurately, and separate the noise from the weak edge. The overall structure of the NSCT is shown in Figure 1(a).  Figure 1(b) illustrates an example of the idealized frequency decomposition.

#### 2.2.2. The Traditional Fuzzy Enhancement Algorithm and Analysis

In light of conventional fuzzy enhancement theory, assume that *I* is a fuzzy matrix, the size of an image *X* is *m* × *n*, and *L* is the grey levels of the image; then *I* can be described as follows:

(1) $$\begin{array}{c} I=\overset{M}{\underset{m=1}{⋃}}\overset{N}{\underset{n=1}{⋃}}\frac{\mu_{mn}}{x_{mn}}, \end{array}$$

where *m* = 1,2,…, *M*, *n* = 1,2,…, *N*. *x*_*mn*_ is the intensity of (*m*, *n*)th pixel. *μ*_*mn*_ denotes the membership value of *x*_*mn*_. In Pal and King's fuzzy enhancement algorithm, the membership function is described as follows:

(2) $$\begin{array}{c} \mu_{mn}=G\left(\;x_{mn}\;\right)={\left[\;1+\frac{\left(L-1\right)-x_{mn}}{F_{d}}\;\right]}^{-F_{e}}, \end{array}$$

where *L* is the maximum gray level of the image *X*. *F*_*d*_ and *F*_*e*_ are related to *μ*_*mn*_ and denote the reciprocal factor and exponent factor, respectively. When *F*_*e*_ = 2, *μ*_*mn*_ = *μ*_*c*_ = *G*(*x*_*c*_), *x*_*c*_ it is called the crossover point. Another fuzzy set is produced by recursion calling as follows:

(3) $$\begin{array}{c} \mu_{mn}^{\prime }=T_{r}\left(\;\mu_{mn}\;\right)=T_{r}\left(\;T_{r-1}\left(\;\mu_{mn}\;\right)\;\right)\;r=1,2,\ldots , \end{array}$$

where *T*_*r*_(*μ*_*mn*_) is defined as successive applications and can be expressed as follows:

(4) $$\begin{array}{c} T_{r}\left(\;\mu_{mn}\;\right)=\left\{\begin{array}{cc} 2{\left(\mu_{mn}\right)}^{2}, & 0\leq \mu_{mn}\leq 0.5\\ 1-2{\left(1-\mu_{mn}\right)}^{2}, & 0.5\leq \mu_{mn}\leq 1. \end{array}\right. \end{array}$$

Finally, (*m*, *n*) pixel value in the enhanced image is obtained by *x*_*mn*_′ = *G*^−1^(*μ*_*mn*_′).

However, there are two faults in Pal and King's method. One is the crossover point that is fixed at 0.5, making it difficult to adapt to the various images. The other is the membership function changes from [*μ*_min_, 1] rather than [0,1], which will lose the image information of the low gray value area after the inverse transformation [17].

Local contrast is based on calculating gray level difference between a specified pixel and its neighboring pixel [19]. According to Thakur and Mishra [20], the fuzzy contrast can be defined as follows:

(5) $$\begin{array}{c} F_{c}\left(\;m,n\;\right)=\left|\;\frac{\mu_{mn}-{\overset{-}{\mu }}_{mn}}{\mu_{mn}+{\overset{-}{\mu }}_{mn}}\;\right|, \end{array}$$

where ${\overset{-}{\mu }}_{mn}$ is the average value of a neighboring window without a center pixel.

### 2.3. The Proposed Method

#### 2.3.1. The Enhancement of Low-Frequency Subband

The low-frequency subband of the original image decomposed by NSCT contains the basic information of the image and includes less noise, while the portion of the basic information relates to the overall contrast of the image. In order to effectively improve the contrast of the image, it is necessary to linearly stretch the low-frequency subband. This is done by first computing the greyscale value of the minimum *x*_min_ and the maximum *x*_max_ and then modifying the grayscale range from (*x*_min_, *x*_max_) to (0,255) by a linear mapping function. The mapping function is as follows:

(6) $$\begin{array}{c} y=f\left(\;x\;\right)=255\times \frac{\left(x-x_{min}\right)}{\left(x_{max}-x_{min}\right)}. \end{array}$$

#### 2.3.2. Denoising in High-Frequency Subband

Part of the high-frequency subband contains the edge details and noises of the original image; it can be divided into three categories: strong edge, weak edge, and noise. Modifying the coefficients of the high-frequency subband retains the strong edge, highlights the weak edge, and suppresses noise effectively. In order to remove noise and retain more details of high-frequency subbands, an adaptive threshold based on Bayes Shrink is used for image denoising [21]. The Bayes Shrink threshold is computed as follows:

(7) $$\begin{array}{c} T_{B}=c\frac{\sigma^{2}}{\sigma_{x}^{2}}, \end{array}$$

where *c* is a constant between 0 and 1. In this paper, *c* is taken as 0.8. *σ*^2^ and *σ*_*x*_^2^ are the noise variance and the signal variance, respectively. Here, a robust median operator is used for calculating the noise variance *σ*

(8) σ=mediandi,jl,k0.6745,σx=max⁡sl,k−σ2,0,sl,k=1mn∑i=1m ∑j=1ndl,k2i,j,

where *s*_*l*,*k*_ denotes the mean square value of the subband coefficient in *l*th level and *k*th direction. *m* and *n* represent the size of the image and *d*_*i*,*j*_(*l*, *k*) is the pixel value at (*i*, *j*) in *l*th level and *k*th direction.

The average *s*_*l*_ of each level is introduced as follows:

(9) $$\begin{array}{c} s_{l}=\frac{\left(\sum_{k=1}^{n}s_{l,k}\right)}{2^{l}}. \end{array}$$

The weighted factors in different directions are computed by the following equation:

(10) $$\begin{array}{c} \lambda =\frac{s_{l}}{s_{l,k}}. \end{array}$$

Adaptive threshold in this study is calculated as follows:

(11) $$\begin{array}{c} T=\lambda T_{B}. \end{array}$$

We adopt the priority threshold *T* to suppress noise.

(12) $$\begin{array}{c} d_{i,j}^{\prime }=\left\{\begin{array}{cc} d_{i,j}, & d_{i,j}>T\\ 0, & d_{i,j}<T, \end{array}\right. \end{array}$$

where *d*_*i*,*j*_′ is the coefficient after denoising.

#### 2.3.3. The Improved Fuzzy Contrast Function and Fuzzy Enhancement Operator

In this paper, the reconstructed image is mapped to the fuzzy domain using the following equation:

(13) $$\begin{array}{c} u_{i,j}=\frac{x_{i,j}-x_{min}}{x_{max}-x_{min}}. \end{array}$$

In (13) *x*_*i*,*j*_ is the reconstructed image pixel value and *x*_min_ and *x*_max_ are the maximum and minimum value of the reconstruct image.

The improved fuzzy contrast can be defined as follows:

(14) $$\begin{array}{c} F=\frac{\left|u_{i,j}-r\right|}{\left|u_{i,j}+r\right|}. \end{array}$$

Then, a nonlinear transformation can be applied to *F*: *F*′ = *ψ*(*F*).  

Ψ(*F*) is a convex transformation that results in *ψ*(0) = 0, *ψ*(1) = 1. The nonlinear enhancement function used in this paper is given below:

(15) $$\begin{array}{c} \psi \left(\;x\;\right)=\frac{1-e^{-kx}}{1-e^{-k}}, \end{array}$$

where *k* is an adjustment factor of the nonlinear enhancement, which takes the value of 1.

The adjusted membership function is

(16) $$\begin{array}{c} u_{i,j}^{\prime }=\left\{\begin{array}{cc} u_{i,j}\left(1-F^{\prime }\right) & u_{i,j}\leq r\\ 1-\left(1-u_{i,j}\right)\left(1-F^{\prime }\right) & u_{i,j}\geq r. \end{array}\right. \end{array}$$

Finally, transfer *u*_*i*,*j*_′ in the fuzzy domain to the spatial domain using the following formula:

(17) $$\begin{array}{c} x_{i,j}^{\prime }=u_{i,j}^{\prime }\left(\;x_{max}-x_{min}\;\right)+x_{min}. \end{array}$$

**Figure 2:**
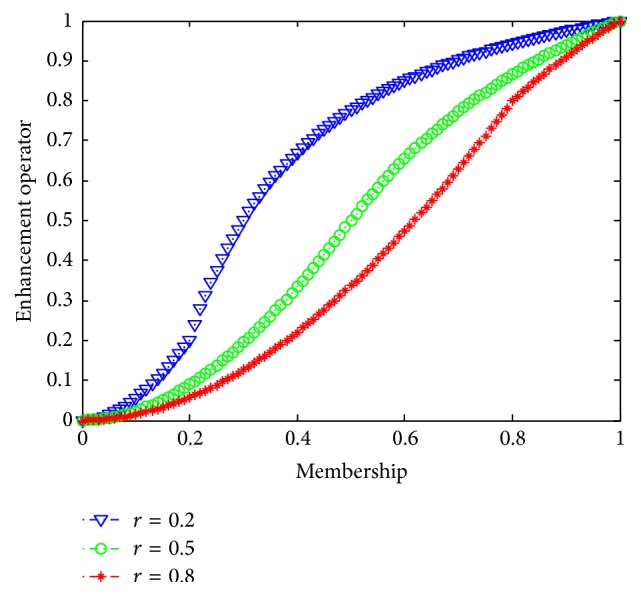
The curve shape of the fuzzy contrast enhancement when *r* takes values as 0.2, 0.5, and 0.8.

In (14) and (16), *r* is an adapted operator from 0 to 1. The different values of *r* represent different fuzzy contrast enhancement curves adapted to different images [22].  Figure 2 shows the shape of the curve when *r* take values of 0.2, 0.5, and 0.8.

**Figure 3:**
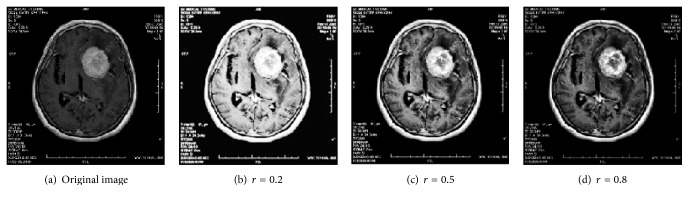
The processing results of a same image when* r,* respectively, take values of 0.2, 0.5, and 0.8.

In general, the detail area is dominated by high gray scale, and the background field is dominated by low gray scale. Experiments have found that when* r* is too small, the background area is overenhanced which results in a reduction of the definition and the overall contrast of the image; when *r* is too big, the detail area is overenhanced which influences the visual effects. Therefore, in this paper, we take *r* within the range of 0.4 to 0.6.  Figure 3 shows the processing results of the same image when *r* takes values of 0.2, 0.5, and 0.8.

#### 2.3.4. Description of the Proposed Algorithm

1. Process the input image based on NSCT, obtaining the low-frequency subband and several high-frequency subbands.
2. Use (6) to obtain linear enhancement. The adaptive threshold process is used for high-frequency subbands to suppress image noise through (7)–(12).
3. Reconstruct the enhanced image from the modified coefficient of low-frequency subband and high-frequency subband by inverse transformation of NSCT.
4. Adopt the improved fuzzy contrast enhancement method to enhance the overall contrast of the image with formulas (13)–(17). Employ the Laplace operator to enhance the details of the image.

## 3. Results and Discussion

**Figure 4:**
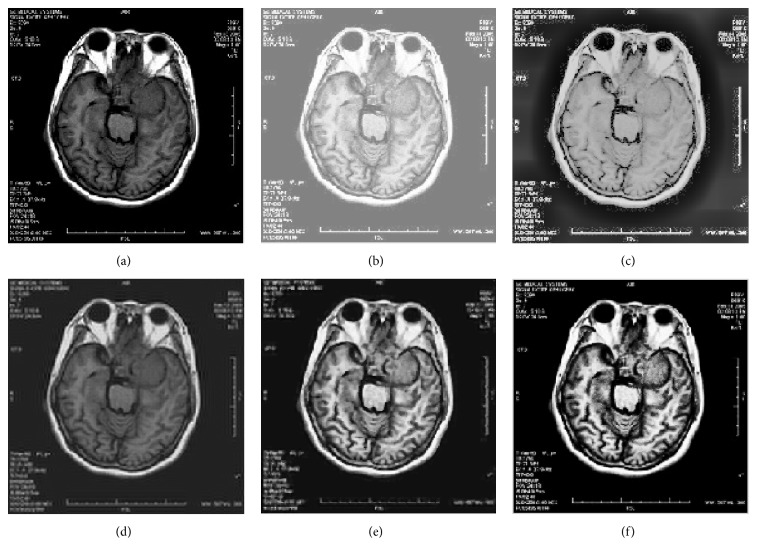
Five methods of enhancing medical images. (a) Original image, (b) enhanced with HE, (c) enhanced with MSR, (d) enhanced with contourlet transform, (e) enhanced with NSCT-FU, and (f) enhanced with the proposed method.

**Figure 5:**
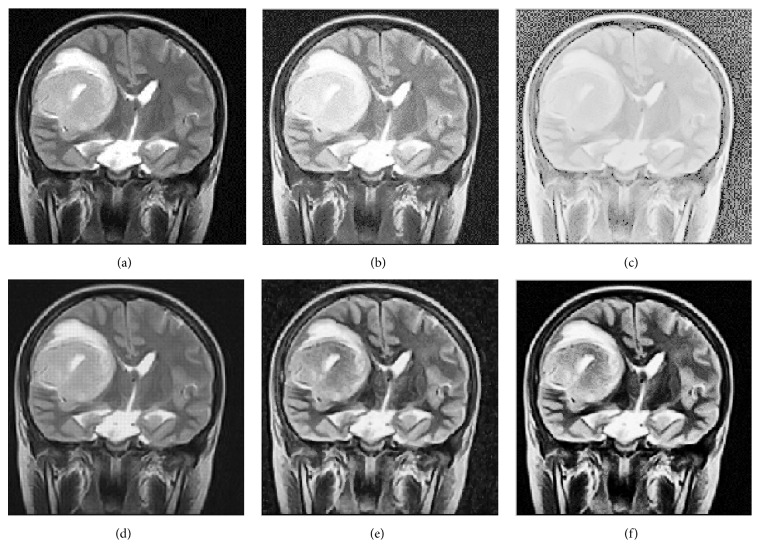
Five methods of enhancing medical images. (a) Original image, (b) enhanced with HE, (c) enhanced with MSR, (d) enhanced with contourlet transform, (e) enhanced with NSCT-FU, and (f) enhanced with the proposed method.

**Figure 6:**
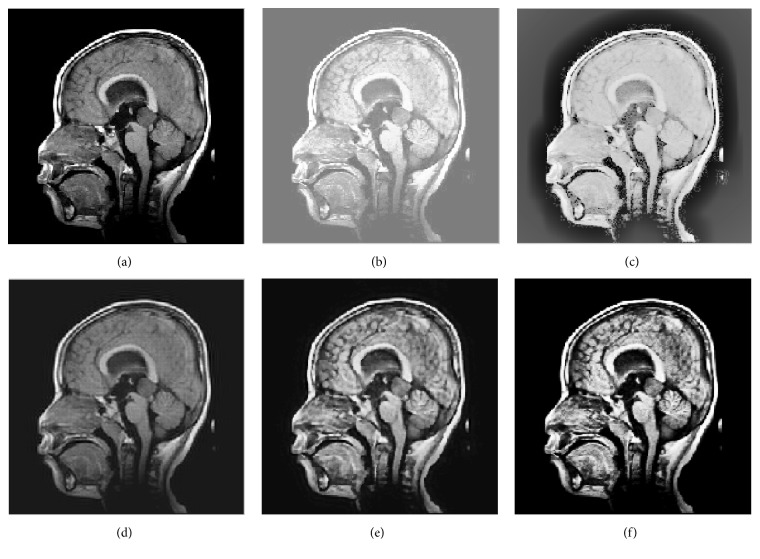
Five methods of enhancing medical images. (a) Original image, (b) enhanced with HE, (c) enhanced with MSR, (d) enhanced with contourlet transform, (e) enhanced with NSCT-FU, and (f) enhanced with the proposed method.

**Figure 7:**
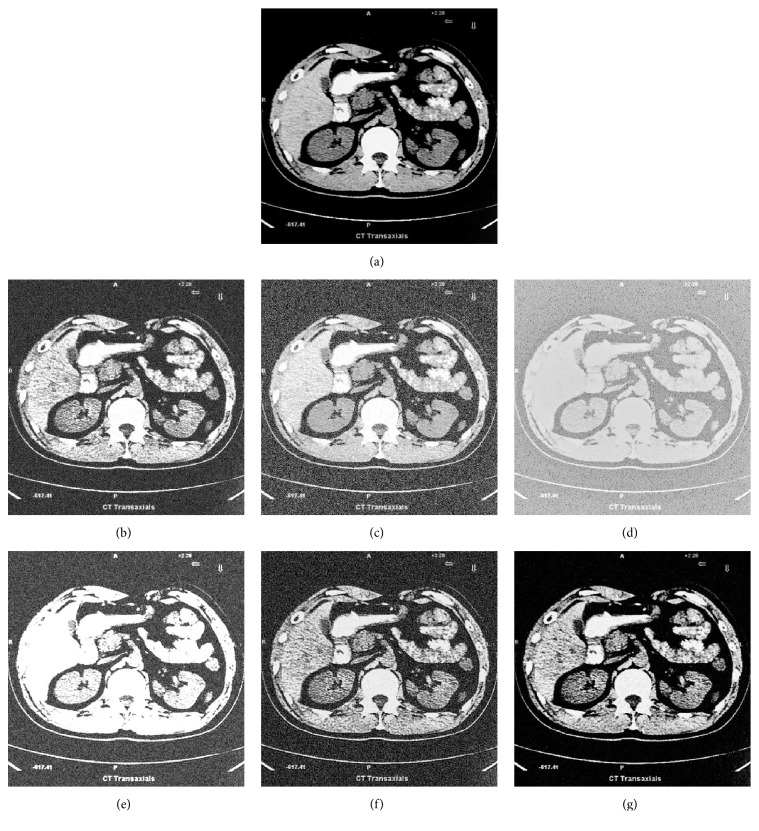
(a) Original image, (b) noisy image with noise level *σ* = 0.2, (c) noisy image enhanced by HE, (d) noisy image enhanced by MSR, (e) noisy image enhanced by contourlet transform, (f) noisy image enhanced by NSCT-FU, and (g) noisy image enhanced by proposed method.

65 medical images were used to evaluate the efficacy of the proposed method. The NSCT decomposition level is three in these experiments. We use 8, 16, and 16 directions in the scales from coarser to finer. In this experiment, we present results obtained by the HE method, multiscale retinex (MSR) [6], contourlet transform [9], improved fuzzy contrast enhancement based on NSCT [17], and the proposed method to verify the effectiveness of the proposed method. Both qualitative and quantitative methods are used to demonstrate the enhanced performance of our proposed approach. Four sets of medical pictures are chosen for samples, and we give the results of the comparative trial and the proposed method when *r* takes values as 0.45 in Figure 4, 0.5 in Figure 5, 0.55 in Figure 6, and 0.5 in Figure 7.

We select information entropy (*H*), structural similarity index measurement (SSIM), the peak signal to noise ratio (PSNR), mean absolute error (MAE), and time as objective evaluation criteria. The entropy represents the overall information of the image, with higher entropy resulting in the image containing more information. It can be defined as follows:

(18) $$\begin{array}{c} H=-\overset{M}{\underset{i=1}{\sum }}\;\overset{N}{\underset{j=1}{\sum }}p\left(\;i,j\;\right)\log p\left(\;i,j\;\right), \end{array}$$

where *M* × *N* is the size of the enhanced image.

SSIM reflects the degree of distortion of the enhanced image. When the SSIM is larger, the image is more clearly defined. It can be defined as follows:

(19) $$\begin{array}{c} SSIM\left(\;x,y\;\right)=\frac{\left(2\mu_{x}\mu_{y}+c_{1}\right)\left(\alpha_{xy}+c_{2}\right)}{\left(\mu_{x}^{2}+\alpha_{x}^{2}+c_{1}\right)\left(\mu_{x}^{2}+\alpha_{x}^{2}+c_{2}\right)}, \end{array}$$

where *μ* is the estimated mean intensity of the image, *α* is the correlation coefficient, and *c*_1_ and *c*_2_ are constants derived from the dynamic range of the images.

The peak signal to noise ratio denotes the denoising performance of the algorithm. When the PSNR is higher, the antinoise performance of the algorithm is better. It can be defined as

(20) $$\begin{array}{c} PSNR=20\times \log\left(\;\frac{L_{D}}{MSE}\;\right), \end{array}$$

where *L*_*D*_ is the maximum value of the pixel and MSE can be defined as follows:

(21) $$\begin{array}{c} MSE=\frac{1}{MN}\overset{M}{\underset{i=1}{\sum }}\;\overset{N}{\underset{j=1}{\sum }}\left[\;{\left(\;x^{\prime }\left(i,j\right)-x\left(i,j\right)\;\right)}^{2}\;\right], \end{array}$$

where *x*′(*i*, *j*) and *x*(*i*, *j*) are the enhanced image pixel and the original image pixel, respectively. MAE denotes the contrast of the enhanced image [23]. A lower MAE results in better contrast. It is computed between the original image and the enhanced image and can be defined as

(22) $$\begin{array}{c} MAE=\frac{1}{M\times N}\overset{M}{\underset{i=1}{\sum }}\;\overset{N}{\underset{j=1}{\sum }}\left|\;x\left(\;i,j\;\right)-x^{\prime }\left(\;i,j\;\right)\;\right|. \end{array}$$

Tables 1–3 show the information entropy, PSNR, MAE, SSIM, and time of the proposed method and the comparative methods. A comparison of the data in the tables shows that even though the SSIM of the medical MRI image enhanced by contourlet transformation is slightly higher than the proposed method in Table 3, the entropy and PSNR are lower in these methods than in the proposed method. On the whole, the SSIM of contourlet transformation is higher than with HE, MSR, and NSCT-FU, but lower than the proposed method. The PSNR of the proposed method is close to NSCT-FU, while it is significantly higher than the other three algorithms. The MAE of the proposed method is lower than in the other four methods, which demonstrates that the proposed algorithm can obtain better contrast than the other algorithms. Although the proposed method has a slower processing time than the other algorithms, it is faster than NSCT-FU. We can analyze the enhanced results objectively using the four sets of pictures used in the above experiments. As can be seen in the enhanced images, the HE method can improve the contrast of images globally; however, it does not take into account relationships among neighborhoods and leads to poor visual effects. At the same time, some details are submerged in the process of enhancement. The contrast enhancement is not obvious in the enhancement results of contourlet transformation, especially in cases where the input image yields low brightness. MSR can improve the luminance of an image effectively while the enhanced image faces overenhancement and noise amplification, and the entropy becomes very low after processing. The enhanced effect of NSCT-FU is close to the proposed method but its definition and contrast are not better than the proposed method.

The average value of 65 medical images is shown in Table 4. To analyze the effectiveness of the proposed method in medical image denoising, a white Gaussian noise with various standard deviations has been added to the experimental images. The experimental results are shown in Figure 7, and the PSNR and SSIM of the different methods are given in Tables 5 and 6. The effectiveness of the proposed method is further demonstrated. When analyzed using both quantitative and qualitative methods, the proposed method has a better effect than the four other enhancement methods.

## 4. Conclusion

This paper proposed an algorithm based on NSCT combining adaptive threshold and improved fuzzy set that follows the demands for medical image enhancement. We improve the fuzzy contrast function and change its normal inverse. At the same time, a new function is designed which can calculate the enhanced pixel gray membership, which is then combined with the Laplace operator to enhance the details. Experimental results show that the proposed method is able to highlight the details and contours of an image, enhance the general contrast of an image, and significantly improve an image's visual effect. Further studies should focus on the adaptability of the algorithm.

## Figures and Tables

**Table 1:** Comparison of different methods using the objective index in Figure 4.

Evaluation standard	Entropy	PSNR	MAE	SSIM	Time
HE	3.44	24.22	0.54	0.294	0.71
MSR	0.00419	24.36	0.24	0.323	7.76
Contourlet	2.02	16.00	0.22	0.753	10.40
NSCT-FU	4.58	25.03	0.08	0.642	43.06
Proposed method	5.82	27.32	0.01	0.836	35.58

**Table 2:** Comparison of different methods using the objective index in Figure 5.

Evaluation standard	Entropy	PSNR	MAE	SSIM	Time
HE	5.75	24.20	0.09	0.682	0.74
MSR	0.000532	24.28	0.40	0.483	8.16
Contourlet	0.10	12.98	0.32	0.747	10.39
NSCT-FU	6.80	26.99	0.05	0.691	44.43
Proposed method	7.23	29.57	0.02	0.875	39.85

**Table 3:** Comparison of different methods using the objective index in Figure 6.

Evaluation standard	Entropy	PSNR	MAE	SSIM	Time
HE	3.51	24.14	0.49	0.356	0.76
MSR	0.002395	24.19	0.34	0.311	8.43
Contourlet	1.94	15.87	0.14	0.776	10.37
NSCT-FU	4.77	25.83	0.07	0.611	44.11
Proposed method	5.97	28.20	0.01	0.713	39.91

**Table 4:** Comparison of different methods using the average objective index of 65 medical images.

Evaluation standard	Entropy	PSNR	MAE	SSIM	Time
HE	4.49	24.64	0.30	0.312	0.79
MSR	0.002236	24.35	0.26	0.365	9.76
Contourlet	1.15	15.87	0.21	0.753	10.51
NSCT-FU	5.66	27.02	0.09	0.635	91.31
Proposed method	6.37	27.20	0.04	0.862	61.20

**Table 5:** PSNR values for Figure 7(a) with the different noise level of the different algorithms.

*σ*_*N*_	0.1	0.2	0.3	0.4	0.5
HE	24.71	23.31	22.65	21.21	20.11
MSR	25.67	25.05	24.61	24.31	23.82
Contourlet	12.03	11.03	10.15	9.40	8.47
NSCT-FU	19.07	17.67	14.85	13.67	12.58
Proposed method	28.02	27.42	27.00	25.92	25.03

**Table 6:** SSIM values for Figure 7(a) with the different noise level of the different algorithms.

*σ*_*N*_	0.1	0.2	0.3	0.4	0.5
HE	0.67	0.61	0.57	0.50	0.45
MSR	0.24	0.21	0.18	0.15	0.11
Contourlet	0.56	0.46	0.45	0.43	0.40
NSCT-FU	0.59	0.55	0.50	0.46	0.42
Proposed method	0.75	0.72	0.68	0.66	0.63
